# The Potential Risk of Plant-Virus Disease Initiation by Infected Tomatoes

**DOI:** 10.3390/plants9050623

**Published:** 2020-05-14

**Authors:** Chen Klap, Neta Luria, Elisheva Smith, Elena Bakelman, Eduard Belausov, Orly Laskar, Oded Lachman, Amit Gal-On, Aviv Dombrovsky

**Affiliations:** 1 Department of Plant Pathology and Weed Research, Agricultural Research Organization, The Volcani Center, 68 HaMaccabim Road, P.O.B 15159 Rishon LeZion 7505101, Israel; chenklap@gmail.com (C.K.); neta.luria8@gmail.com (N.L.); elishevasmith@gmail.com (E.S.); elenab@volcani.agri.gov.il (E.B.); odedl@volcani.agri.gov.il (O.L.); amitg@volcani.agri.gov.il (A.G.-O.); 2 The Robert H. Smith Faculty of Agriculture, Food and Environment, The Hebrew University of Jerusalem, Rehovot 761001, Israel; 3 Department of Ornamental Plants and Agricultural Biotechnology, Agricultural Research Organization, The Volcani Center, 68 HaMaccabim Road, P.O.B 15159 Rishon LeZion 7505101, Israel; eddy@volcani.agri.gov.il; 4 Israel Institute for Biological Research (IIBR), Ness-Ziona 74100, Israel; orlyl@iibr.gov.il

**Keywords:** primary viral inoculum, mechanical transmission, tomato brown rugose fruit virus, pepino mosaic virus

## Abstract

During 2019, tomato fruits showing viral-like symptoms of marbled yellow spots were abundant in Israel. The new symptoms were distinctive from those typical of tomato brown rugose fruit virus (ToBRFV) infection but resembled symptoms of pepino mosaic virus (PepMV) infection. RT-PCR analysis and the serological tests (enzyme linked immunosorbent assay, western blot and in situ immunofluorescence) revealed and confirmed the presence of both the tobamovirus ToBRFV and the potexvirus PepMV in the symptomatic fruits. A mixture of rod-like and filamentous particles, characteristic of viruses belonging to tobamovirus and potexvirus genera, was visualized by transmission electron microscopy of the tomato fruit viral extract. Sanger sequencing of amplified PepMV-coat protein gene segments showed ~98% sequence identity to the Chilean (CH2)-strain. In a biological assay testing the contribution of traded infected tomatoes to the establishment of tomato plant disease, we applied direct and indirect inoculation modes using *Tm-2^2^*-resistant tomato plants. The results, assessed by disease symptom development along with serological and molecular analyses, showed that the ToBRFV and PepMV co-infected fruits were an effective inoculum source for disease spread only when fruits were damaged. Importantly, intact fruits did not spread the viral disease. These results added a new factor to disease epidemiology of these viruses.

## 1. Introduction

Tomato (*Solanum lycopersicum* L.) crop production is hindered by viral diseases worldwide. Recently, pest alerts have been issued in several countries regarding the spread risk of the tobamovirus tomato brown rugose fruit virus (ToBRFV), identified in Jordan and Israel [1,2], causing severe damages to tomato production in the Middle East. According to the European and Mediterranean Plant Protection Organization (EPPO) global database https://gd.eppo.int/taxon/tobrfv, ToBRFV has been detected recently in Europe [3,4,5], North America [6,7] and China [8]. It is too early to estimate global crop production damage, but from data gathered in Florida, USA, for example, 30%–70% yield loss was estimated for the state tomato crop, which accounts for $262 million USD a year economic impact (https://news.wgcu.org/post/virus-found-mexican-tomatoes-worries-florida-agriculture-officials). ToBRFV is a single-stranded positive-sense RNA virus of ~6.4 kb encapsulated in a rod-like particle ~300 nm long. The virus encodes the four well-known viral proteins: two comprising the RNA-dependent RNA polymerase (RdRP) complex, a movement protein and a ~17 kD coat protein (CP). ToBRFV causes a range of symptoms in tomatoes harboring the *Tm-2^2^* resistance allele. Fruit yellowing and bleaching are the most commonly occurring symptoms, often accompanied by a necrotic peduncle [2,9].

In the last two decades, pepino mosaic virus (PepMV) has been a major concern for tomato growers globally, especially in Europe and North America [10,11,12,13,14,15,16], according to the EPPO global database (https://gd.eppo.int/taxon/PEPMV0). PepMV can cause severe damages, resulting, for example, in an estimated 50%–60% unmarketable fruits in Belgium (http://pmv-01.com/en/). PepMV belongs to the *Potexvirus* genus of the *Flexiviridae* family. The virions contain a positive-sense single-stranded RNA genome of 6.4 kb encapsidated in filamentous particles 508 nm long [17]. The RNA genome is comprised of five open reading frames encoding the RdRP, a triple gene block and a ~26 kD CP [18]. PepMV infection of tomato plants is associated with a wide range of symptoms including marbled fruits [19]. Multiple factors are involved in symptom development, including environmental conditions, viral accumulation, tomato cultivar and growth stage [20]. Four major PepMV genotypes were identified in America and Europe: the Peruvian genotype (LP), discovered in pepino (*Solanum muricatum*), the European genotype (EU), first discovered in tomatoes (*Solanum lycopersicum*), the Chilean genotype (CH2) commonly occurring in Europe, and the American genotype (US1) [11,17,21,22,23,24,25]. Although no correlation was observed between the various genotypes and the symptom phenotype [20], specific severe symptoms could be attributed to differences in the nucleotide sequence of isolates from the same genotype [25,26,27,28,29,30].

Similar to ToBRFV [2], the PepMV disease is primarily transmitted via mechanical plant handling. Regarding PepMV, even direct contact between healthy and infected plants can spread the disease [17]. Beneficial insects were also implicated in both PepMV and ToBRFV disease spread [31,32]. The potexvirus PepMV and tobamoviruses in general are considered stable seed-borne viruses [33], although the seed-to-seedling transmission ratio of each virus family is low [34,35]. Virus-infected seeds could constitute a primary source of infection primarily via plant transplantation. Here, we report a recent detection of PePMV in Israel in mixed infections with ToBRFV in traded tomatoes, and have analyzed the risks of viral disease initiation by the co-infected fruits.

## 2. Results

### 2.1. Locally Distributed Tomatoes Showing Viral-Like Disease Symptoms Were Co-Infected by ToBRFV and PepMV

**Figure 1:**
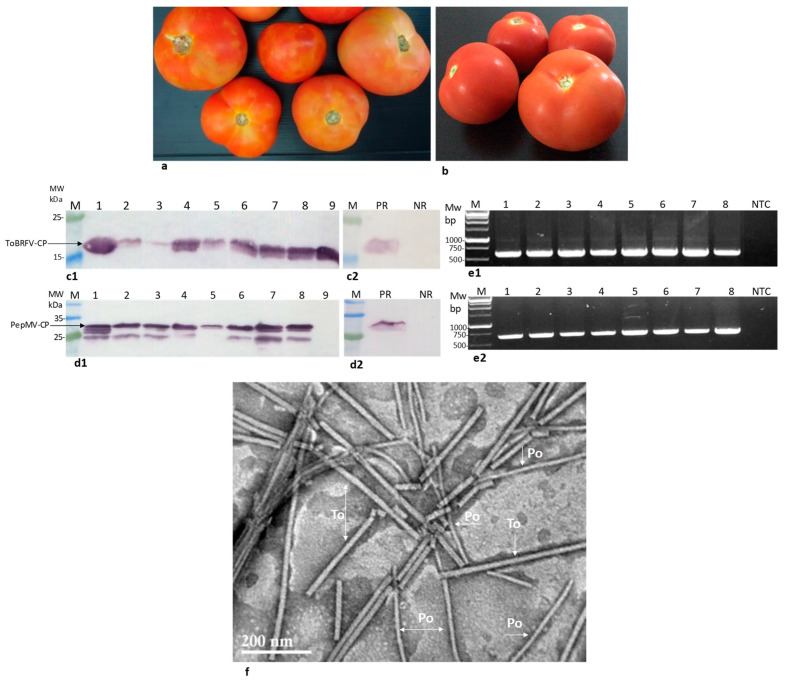
Symptomatic traded tomatoes were co-infected by tomato brown rugose fruit virus (ToBRFV) and pepino mosaic virus (PepMV). (**a**) Symptomatic tomatoes showing bleaching and marbled yellow spot phenotypes; (**b**) Asymptomatic tomatoes; (**c**,**d**) Western blot analyses of symptomatic tomatoes; (**c1**) ToBRFV coat protein (CP) detection in symptomatic fruits; (**c2**) CP detection in ToBRFV-infected tomato leaves; (**d1**) PepMV CP detection in symptomatic fruits. The extra low molecular weight bands detected by the antibodies are apparently degradation products, as compared with data presented in (**d2**); (**d2**) CP detection in PepMV-infected tomato leaves; (**e1**) RT-PCR amplified 615 bp segments of ToBRFV CP in the western blot positive co-infected tomatoes; (**e2**) RT-PCR amplified 650 bp segments of PepMV CP in the western blot positive co-infected tomatoes; (**f**) Transmission electron microscopy visualization of symptomatic fruit virion purification showing particle morphology resembling tobamovirus and potexvirus viral genera. M, molecular weight; PR, positive reference; NR, negative reference; NTC, non-template control; Po, potexvirus; To, tobamovirus; arrows mark specific viral particles.

**Figure 2:**
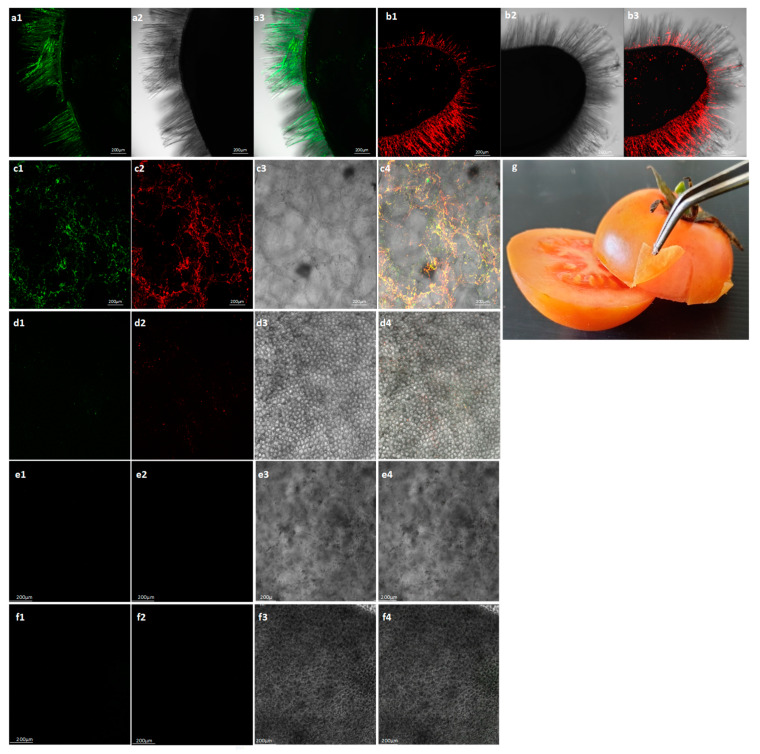
In situ immunofluorescence localization of tomato brown rugose fruit virus (ToBRFV) and pepino mosaic virus (PepMV) in symptomatic tomatoes. (**a1**) ToBRFV coat protein (CP) detection in infected tomato seeds using antibodies conjugated to Alexa 488, visualized with green channel; (**a2**) ToBRFV-infected tomato seeds visualized using bright field; (**a3**) ToBRFV-infected tomato seeds visualized by merging channels; (**b1**) PepMV CP detection in tomato seeds using antibodies conjugated to Alexa 594, visualized with red channel; (**b2**) PepMV-infected tomato seeds visualized using bright field; (**b3**) PepMV-infected tomato seeds visualized by merging channels; (**c1**) ToBRFV-CP detection in co-infected tomato exocarp, facing the mesocarp, using the green channel; (**c2**) PepMV-CP detection in the co-infected tomato exocarp, facing the mesocarp, using the red channel; (**c3**) co-infected tomato exocarp, facing the mesocarp, visualized using bright field; (**c4**) ToBRFV and PepMV detection in co-infected tomato exocarp, facing the mesocarp, using merged channels; (**d1**) ToBRFV-CP detection in co-infected tomato exocarp surface using the green channel; (**d2**) PepMV-CP detection in co-infected tomato exocarp surface using the red channel; (**d3**) Co-infected tomato exocarp surface visualized using bright field; (**d4**) Exocarp surface from a co-infected tomato visualized using merged channels; (**e**,**f**) Exocarp dissected from a control healthy tomato subjected to immunofluorescence. (**e1**) Imaging of exocarp facing the mesocarp using the green channel; (**e2**) Imaging of exocarp facing the mesocarp using the red channel; (**e3**) Imaging of exocarp facing the mesocarp using bright field; (**e4**) Imaging of exocarp facing the mesocarp using merged channels; (**f1**) Imaging of exocarp surface using the green channel; (**f2**) Imaging of exocarp surface using the red channel; (**f3**) Imaging of exocarp surface using bright field; (**f4**) Imaging of exocarp surface using merged channels; (**g**) Exocarp dissection from a co-infected tomato fruit.

Symptomatic traded tomatoes showing marbled yellow spots were observed during 2019 (Figure 1a,b). The sampled fruits were subjected to an enzyme linked immunosorbent assay (ELISA) test showing mixed infections of ToBRFV and PepMV in three of the presented symptomatic fruits. Optical density (O.D.) values were in the range of 0.10–2.28 for ToBRFV-positive tomatoes and 1.88–3.35 for PepMV-positive fruits, while the negative control values were 0.02 and 0.03, respectively. Western blot analyses of the viral CPs in an additional eight symptomatic fruits showed the range of ToBRFV and PepMV CP levels in the analyzed fruits (Figure 1c,d). One asymptomatic tomato was positive for ToBRFV only. The symptomatic fruits, western blot-positive for both PepMV and ToBRFV, were also subjected to viral RNA extraction followed by RT-PCR amplification (Figure 1e). The authenticity of the amplicons obtained from ToBRFV and PepMV was confirmed by Sanger sequencing. The obtained PepMV isolates’ sequences were submitted to GenBank (accession Nos. MT018445, MT018446, MT018447, MT018448, MT018449). Importantly, all PepMV-CP amplicons showed ~98% sequence identity to Chilean (CH2) strains (GenBank accession Nos. MN182534, MK133092, MF422615). Virion preparations from five symptomatic fruits subjected to transmission electron microscopy (TEM) analysis showed particles of rod-like and filamentous morphology, characteristic of viral particles belonging to tobamovirus and potexvirus genera (Figure 1f). These results, documenting the new occurrence of PepMV in Israel, prompted our study of the possible differential localization of the two viruses, PepMV and ToBRFV, in the tomato fruits as well as the infectivity potential of the co-infected fruits (see below). Using in situ immunofluorescence to detect the viruses’ CP, we showed that both viruses are located in the seeds of the viral-infected fruits (Figure 2a,b). ToBRFV and PepMV CP visualization in the exocarp internal side of the co-infected fruits showed both viruses (Figure 2(c1,c2)), which were apparently co-localized (Figure 2(c4)). Importantly, the exocarp surface was devoid of both ToBRFV and PepMV (Figure 2d).

### 2.2. Assessment of the Tomato Fruit ToBRFV Infectivity Potential

**Figure 3:**
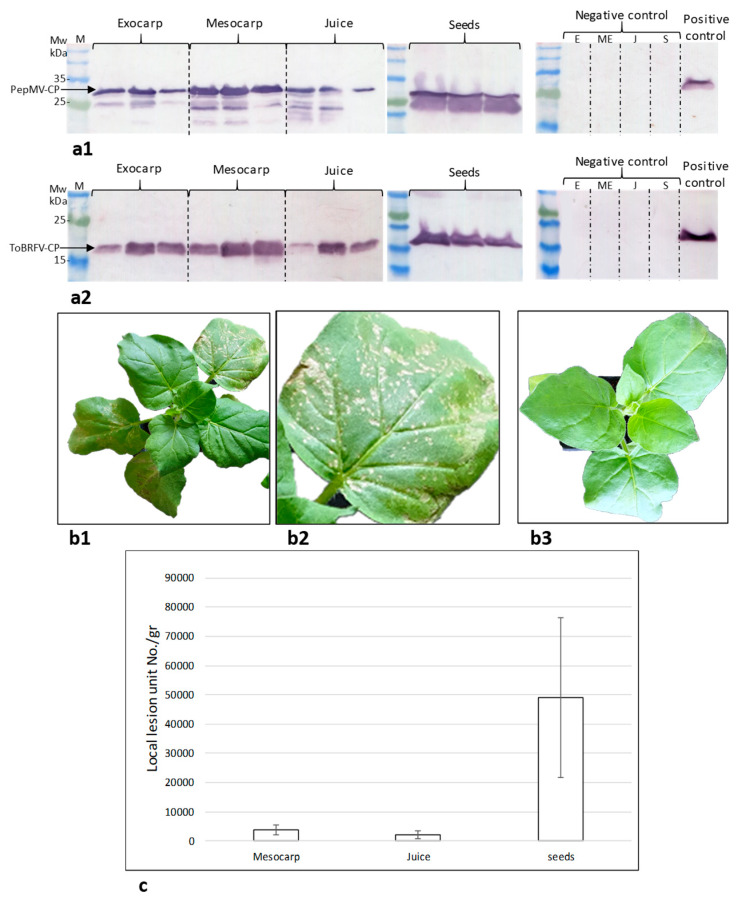
Infectivity potential of components of tomato brown rugose fruit virus (ToBRFV) and pepino mosaic virus (PepMV) co-infected tomatoes. (**a**) Western blot analyses of co-infected tomato fruit components; (**a1**) PepMV coat protein (CP) detection in fruit components of three representative symptomatic fruits; (**a2**) ToBRFV CP detection in fruit components of three representative symptomatic fruits; (**b1**) A biological assay allowing visualization of ToBRFV induced local lesions on a representative *Nicotiana glutinosa* plant; (**b2**) A close-up image of ToBRFV induced local lesions on a *N. glutinosa* leaf; (**b3**) An untreated *N. glutinosa* plant; (**c**) ToBRFV-induced local lesion number on *N. glutinosa* leaves (*n* = 27 for each test), in one gram of fruit mesocarp, juice or seeds; M, molecular weight; E, exocarp; ME, mesocarp; J, juice; S, seeds.

The infectivity potential of the virus-infected tomato fruits could be estimated by quantifying biologically active virus particles using a local lesion assay and/or calculating the ratio of symptomatic tomato plants using an inoculation assay (see below). Importantly, a test plant for PepMV local lesion count has not been reported yet. Unlike PepMV, ToBRFV induces local lesions in tobacco plants. We therefore evaluated the number of ToBRFV induced local lesion units using *Nicotiana glutinosa* as indicator plants. Symptomatic tomato fruit components (exocarp, pericarp, juice and seeds) were subjected to western blot analyses. ToBRFV-CP and PepMV-CP were detected in all tested fruit components (Figure 3(a1,a2)). Mesocarp, juice and seeds of the co-infected fruits were tested for local lesion induction (Figure 3(b1,b2)). The calculated local lesion unit number per gram fruit component was the highest in seeds (Figure 3c).

### 2.3. Symptomatic Co-Infected Tomato Fruits Could Constitute a Source of Tomato Plant Viral Infection

Symptomatic co-infected tomato fruits, Exp. 1–3, were subjected to biological assays testing the possibility that virus-infected-fruits could initiate a disease. An additional batch, Exp. 4, of symptomatic tomato fruits infected with ToBRFV only, commonly observed at high environmental temperatures, was subjected to the biological assay as well. ToBRFV and PepMV infection of the traded fruits was shown using ELISA (Table 1).

Four possible inoculation modes (A–D) were implemented using *cv*. Ikram tomato plants harboring the *Tm-2^2^* resistance allele. A and B inoculation modes tested the infectivity of intact symptomatic fruits while C and D inoculation modes tested the infectivity of symptomatic fruit juice. The study of the inoculated plants was carried out at different temperatures, since it has been reported that the PepMV-CH2 strain is responsive to temperature changes [20] and we have observed that severe ToBRFV symptoms were seasonal. Seven days post-disease symptom development, leaf samples from all the inoculated tomato plants were collected and subjected to ELISA analyses (Table 2). 

**Figure 4:**
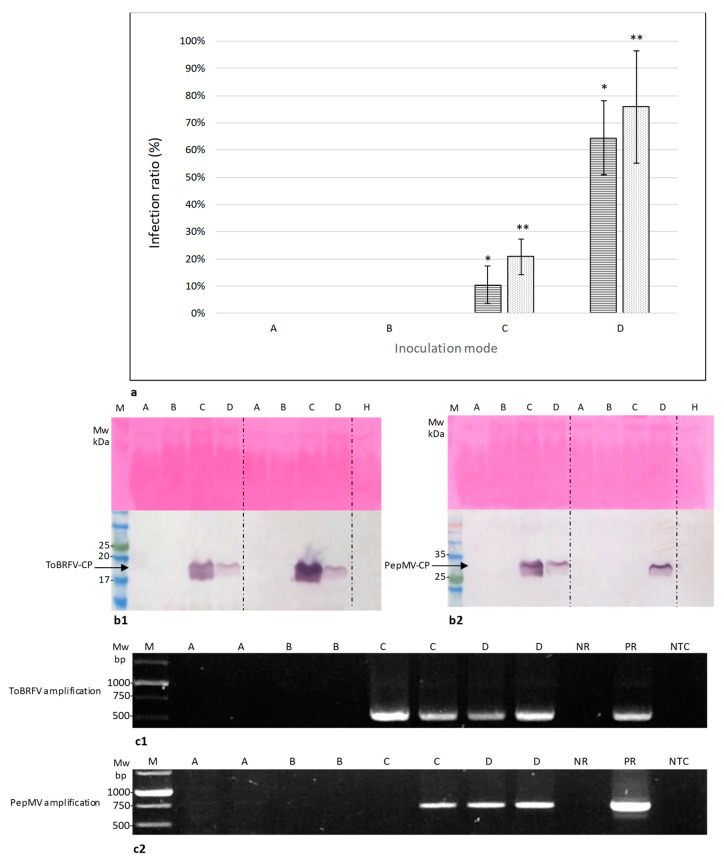
Pepino mosaic virus (PepMV) and tomato brown rugose fruit virus (ToBRFV) infectivity potential of the symptomatic tomatoes. (**a**) A graph of ToBRFV and PepMV infection ratio means ± standard deviations, using A–D inoculation modes, as calculated from exp. 1–4 (presented in Table 2); (**b**) Western blot analyses of tomato plants inoculated by two different fruits using A–D inoculation modes. (**c**) RT-PCR analyses of tomato plants inoculated by two different fruits using A–D inoculation modes. (**c1**) RT-PCR amplified 615bp segments of ToBRFV CP. (**c2**) RT-PCR amplified 650 bp segments of PepMV CP. A, rubbing leaves with intact symptomatic fruits; B, rubbing leaves with hands after touching intact symptomatic fruits; C, applying symptomatic fruit juice onto tomato leaves; D, rubbing leaves with symptomatic fruit juice; M, molecular weight; H, healthy leaves; ToBRFV is marked with dots; PepMV is marked with horizontal lines; * *p* < 0.05 (*p* = 0.0266), *t*-test comparing PepMV inoculation modes C and D; ** *p* < 0.05 (*p* = 0.008), *t*-test comparing ToBRFV inoculation modes C and D. Bars are standard deviation of the mean. H, healthy tomato plants; NR, negative reference; PR, positive reference; NTC, non-template control.

Most importantly, rubbing the intact symptomatic fruits onto the tomato leaves as well as rubbing tomato leaves with hands contaminated by touching the intact symptomatic fruits did not result in viral disease infection of the tomato plants as observed visually (no disease symptoms) and validated by ELISA tests (Table 2, treatments A and B). Unlike the intact symptomatic-tomatoes’ negative results, ToBRFV and PepMV were positively detected in the plants inoculated with symptomatic fruit juice (Table 2, treatments C and D). The ToBRFV and PepMV average percent infectivity rate values (±standard deviations) of symptomatic fruit juice applied directly onto leaves (without leaf rubbing) were 20% ± 6% and 10.48% ± 6%, respectively, while the ToBRFV and PepMV average percent infectivity rate values (± standard deviations) of symptomatic fruit juice applied onto leaves with rubbing were 75% ± 20% and 64% ± 13%, respectively (Figure 4a). Western blot analyses and RT-PCR tests of sampled inoculated leaves confirmed these results (Figure 4b,c)

**Figure 5:**
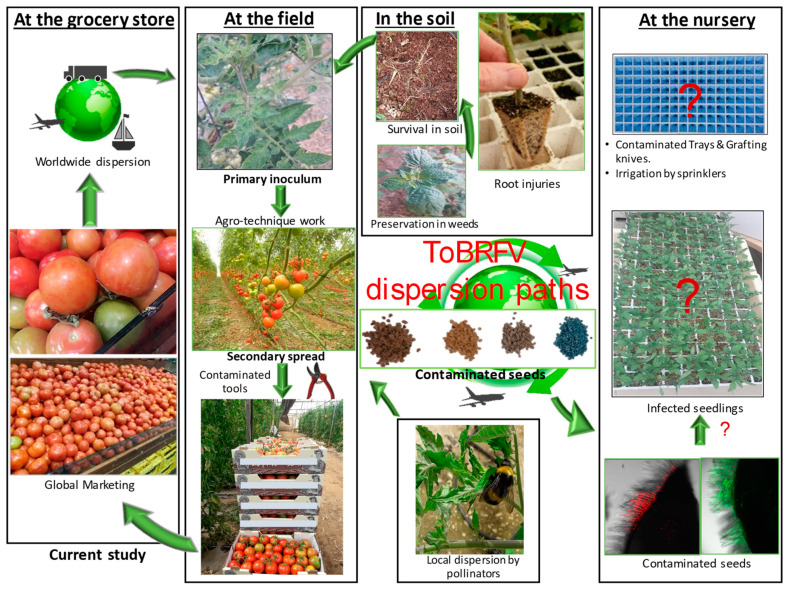
A general scheme illustrating the contribution of the current study to viral disease spread amid the broad epidemiological context of factors involved in tomato brown rugose fruit virus (ToBRFV) disease distribution.

The contribution of ToBRFV-infected tomato juice to the viral disease spread adds an important new factor involved in the epidemiology of ToBRFV, as depicted in Figure 5.

## 3. Discussion

During 2014–2016, ToBRFV disease spread in Israel to most of the greenhouse-grown tomato plants harboring the *Tm-2^2^* resistance allele [2,9]. In recent years, ToBRFV occurrence has been reported in Europe [3,4,5], North America [6,7] and China [8] while PepMV was already established globally [36]. Our study revealed the occurrence of PepMV, which was new to Israel, in mixed infections with ToBRFV in traded tomatoes (Figure 1 and Figure 2). The PepMV CP isolates showed high sequence similarity to PepMV CH2 strains isolated in England, Germany and Switzerland [3,5,37]. Importantly, sequencing of the whole genome, confirmed by biological experiments, are necessary to classify these PepMV isolates as attenuated or mild/aggressive strains.

Global disease spread of mechanically transmitted seed-borne viruses has been often attributed to the worldwide trade in seeds. However, the seed-to-seedling transmission ratio of PepMV is low [34] and we are currently investigating the seed-to-seedling transmission ratio of ToBRFV. To provide insights into the potential risks of viral disease spread originated by infected fruits, constituting a potential inoculum source, we initiated a study using the ToBRFV and PepMV co-infected tomato fruits as a model for risk assessment. Recently, the United States Department of Agriculture (USDA) Animal and Plant Health Inspection Service (APHIS) has issued a Federal Order restricting tomato fruit imports from countries with ToBRFV (https://www.aphis.usda.gov/aphis/ourfocus/planthealth/import-information/federal-import-orders/tobrfv/tomato-brown-rugose-fruit-virus). However, the scientific literature lacks reports of experimental studies on the potential risk of viral disease spread by infected fruits or fruit components, in particular regarding the new risk of ToBRFV distribution. When addressing the question whether symptomatic traded tomatoes could be the source of viral infection, we have shown that seeds, fruit juice, mesocarp and exocarp facing the mesocarp of the symptomatic fruits were infected by both ToBRFV and PepMV (Figure 2 and Figure 3). Interestingly, using in situ immunofluorescence, we have shown that the surface of the fruit exocarp was devoid of the viruses (Figure 2). Importantly, ToBRFV in the symptomatic fruits caused local lesions in *N. glutinosa* plants and the ToBRFV and PepMV mixed-infected fruits could initiate disease spread of both viruses in tomato plants inoculated with the fruit juice (Table 2, Figure 4). The inoculation studies using the co-infected symptomatic fruits were performed in three different experiments conducted at various environmental temperatures (Table 2, Exp. 1–3). Interestingly, the efficiency of ToBRFV infection was the lowest in Exp.2, which was conducted at low daytime temperatures of 16–20 °C and symptoms were apparent only thirty-five days post-inoculation, showing low responsiveness of ToBRFV infection at these temperatures. The results of PepMV infection, however, did not show a clear temperature effect. Most importantly, the results of the in situ immunofluorescence analyses, showing a lack of virus detection on the surface of the symptomatic fruits (Figure 2), were confirmed in all of the biological assays. Rubbing intact fruits onto tomato leaves did not initiate a viral disease during the time span of our study (Table 2, Figure 4).

In our studies, we have observed that symptomatic tomato fruits in systemically ToBRFV-infected plants amounted only to ≤30% of the fruit yield, depending on the plant variety and climate conditions, although all the fruits were ToBRFV-positive (Dombrovsky et al., unpublished data). This implies that a low percentage of symptomatic fruits could still be an indication of a vast disease spread potential. Our study apparently provided a link between traded-infected fruits and a possible viral epidemic in the farms. In a scheme, summarizing ToBRFV dispersion paths (Figure 5), it is suggested that traded fruits could constitute an additional primary source of infection, which was until now attributed mostly to seed trade and to crops or weeds grown in soil contaminated from previous growing cycles. As mentioned above, although ToBRFV is a seed-borne virus and easily detected in contaminated seeds (by in situ immunofluorescence and western blots, Figure 2 and Figure 3), disease spread from seed-to-seedling is still under study. Importantly, there is a major contribution of mechanical transmission to a secondary spread of the virus either via plant manipulations such as pruning, trellising or spread by beneficial pollinators [32]. Practicing hygienic behavior (e.g., while fruit consuming in tomato growing areas), compatible to the outcome of our data, could reduce the risk of viral disease spread. An additional interesting outcome derived from this current study is the application of final-fruit packaging procedures in proximity to the growing areas, thereby preventing fruit manipulations and minimizing the risks of viral disease spread by consumers worldwide.

The various strains of PepMV often occur in mixed infections, which contribute to molecular evolution of the virus [23,37,38]. There are several reports on the occurrence of PepMV in mixed infections with viruses belonging to different genera [3,7,39,40,41]. The contribution of PepMV to disease severity is not always clear [41]. Interestingly, PepMV found in mixed infections with the tomato torrado virus, which causes the torrado disease in tomatoes, was suggested to participate in modulation of the epidemiology of the viruses [40].

The considerable ease of ToBRFV and PepMV disease spread in tomato plants via fruit juice of infected tomatoes, demonstrated in our study, now necessitates a comprehensive study of the epidemiology of the viral disease caused by the co-infecting viruses. Whether the co-infection adds to symptom severity or to the velocity of disease spread is a question that still needs to be addressed.

## 4. Materials and Methods

### 4.1. Tomatoes and Tomato Plant Samples Subjected to Analyses

During 2019, traded tomatoes showing marbled yellow spots were selected for analyses. Fruits were either collected from grocery stores in Rishon LeZion, Rehovot and Tel-Aviv in Central Israel, or from growers in the Besor area in Southern Israel. The symptomatic tomatoes (dissected pericarp as well as seeds, extracted juice, mesocarp and exocarp from a total of sixteen symptomatic fruits) and laboratory-inoculated tomato plants (leaves) obtained from seventy-five symptomatic fruits, were subjected to serological assays (see below) using the antisera specific for ToBRFV [2] and PepMV (AS-1022, kindly provided by Wulf Menzel, Leibniz Institute DSMZ-GmbH, Braunschweig, Germany). Tomatoes (dissected pericarp) and mechanically inoculated tomato plants (leaves) were also subjected to viral RNA extraction and RT-PCR analyses (see below). For local lesion count, fruit components (seeds, juice and mesocarp), extracted from nine tomatoes, were suspended in 10 mM phosphate buffer, pH = 7.0. The seeds were suspended in a ratio of 1 g/10 mL buffer because more concentrated ratios did not show distinct local lesions, while juice and mesocarp were suspended in a ratio of 1 g/mL buffer. An aliquot of fifty µL from each suspension was rubbed onto three leaves of *N. glutinosa* plants, one plant for a fruit. Local lesion unit Nos. in one gram of fruit component was calculated according to the appropriate dilution factor.

### 4.2. Viral RNA Extraction, Reverse Transcription (RT) and PCR Amplification

Tomato fruits (pericarp) and leaf samples of mechanically inoculated tomato plants were ground in general extraction buffer (Bioreba, Reinach, Switzerland). The Accuprep Viral RNA Extraction kit (Bioneer, Daejeon, Korea) was used for viral RNA extraction. For reverse transcription (RT), the qPCRBIO cDNA synthesis kit (PCR Biosystems, London, UK) was used. The obtained cDNA served for PCR amplification using the following designed primer sets: for ToBRFV 615 bp amplicon, F-5557F (5′-TTTAGTAGTAAAAGTGAGAAT-3′) and R-6167R (5′-TTGTAAACCGGATGCACTTTCAAATG-3′) primers were employed, and for PepMV 650 bp amplicon, the primers used were F-5658F (5′-CCATCAGATGCACCACCAAC-3′) and R-6307R (5′-TTAGCTCCTCCCATGTGTCC-3′). Amplicons obtained from selected samples were Sanger sequenced (HyLabs, Rehovot, Israel). The authenticity of each amplified genome segment was confirmed using the Basic Local Alignment Search Tool (BLAST) search algorithm against the National Center for Biotechnology Information (NCBI) GenBank (https://blast.ncbi.nlm.nih.gov/Blast.cgi).

### 4.3. Western Blot Analysis

Samples of symptomatic tomato fruits (pericarp, seeds, juice, mesocarp, exocarp) and mechanically inoculated tomato plants (leaves) were weighed and subjected to protein extraction by suspending the weighed samples in USB buffer containing 75 mMTris-HCL (pH6.8), 8 M urea, 4.5% (*g/v*) SDS and 7.5% (*v/v*) ß-mercaptoethanol while keeping constant 1 µg/5.5 µL ratios in all samples. The extraction was carried out by crushing the fruit samples in the USB buffer, or mixing the fruit juice with the USB buffer and incubating the suspensions at 90 °C for 15 min. The suspensions were centrifuged at 14,000× *g* for 15 min and the supernatants were subjected to western blot analyses as previously described [2]. Samples were separated on 15% SDS-PAGE. The gels were electro-blotted onto a nitrocellulose membrane for 30 min at 200 mAmp (for a single gel) using a semi-dry transfer apparatus (Bio-Rad). The membrane was blocked for 2 h at room temperature with 3% non-fat dry milk in PBS and the specific antisera for ToBRFV or PepMV was added for overnight incubation at 4 °C. The alkaline phosphatase (AP) conjugated goat anti-rabbit antibodies (Sigma) were used for detection with the addition of AP-substrate NBT, BCIP (Bio-Rad). Ponceau-s staining, using 0.2% (*w/v*) Ponceau-s powder prepared in 5% acetic acid in PBS (*v/v*) was conducted after the detection of the viral CP to prevent the Ponceau-s background prior to CP detection. Importantly, in fruit samples (pericarp, mesocarp, exocarp and juice), Ponceau-s staining was not sensitive enough to show the low protein levels.

### 4.4. Indirect Enzyme-Linked Immunosorbent Assay (ELISA)

Tomato fruit samples (calyx, pericarp) and mechanically inoculated tomato plants (leaves) seven days post disease symptom development, were subjected to indirect ELISA test [42]. Samples were ground in coating buffer (Agdia) and were incubated for 3 h at 37 °C with 1:5000 dilution of the ToBRFV antiserum or the PepMV antiserum. Detection was carried out by incubating the samples with AP-conjugated goat anti-rabbit (IgG) (Sigma, Steinheim, Germany) for 3 h at 37 °C. P-nitro phenyl phosphate (Sigma) substrate was used at a concentration of 0.6 mg/mL. The developing color was recorded by ELISA reader (Thermo Fisher Multiskan FC) at 405 nm and 620 nm. O.D. values of a minimum ratio of three times the value of the negative (healthy) controls were considered positive.

### 4.5. Transmission Electron Microscopy (TEM)

Purification of viral particles was performed using 100 g symptomatic tomato fruits (pericarp) crushed in 100 mL 0.1 M potassium phosphate buffer, pH = 7.0 containing 0.5% sodium sulphite. Chloroform-butanol mixture (1:1 *v/v*) comprising 10% of the fruit solution volume was added and the total mixture was incubated 1 h at 4 °C. After centrifugation at 13,000× *g* for 20 min, the supernatant was filtered through Miracloth (Calbiochem) and the filtrate was ultra-centrifuged at 200,000× *g* for 2.5 h. The pellet was suspended in 1 mL 0.01 M potassium phosphate buffer pH = 7.0 and placed on 4 mL sucrose 30% in 0.01 M potassium phosphate buffer pH = 7.0. The final virus preparation was pelleted by ultra-centrifugation at 200,000× *g* for 2.5 h. TEM analysis was performed as previously described [2] using 2% uranyl acetate and visualization using an FEI Tecani T12, equipped with Gatan ES500W Erlangshen camera.

### 4.6. In Situ Immunofluorescence Labelling of ToBRFV and PepMV

Symptomatic tomato fruit exocarp dissected with a sterile razor blade as well as isolated seeds, washed in water for 24 h, were subjected to in situ immunofluorescence tests. Samples were fixed for 1–2 h at room temperature using fixation buffer (adjusted to pH = 7.0, using NaOH) containing 4% (*v/v*) formaldehyde, 0.2% (*v/v*) glutaraldehyde, 50 mM 1,4-piperazinediethanesulfonic acid (PIPES) and 1 mM CaCl_2_, as described previously [43]. Samples were washed twice with phosphate-buffered saline (PBS) solution containing 0.05% (*w/v*) Tween-20 (PBS-T). Blocking was performed with PBS containing 1% (*w/v*) skim milk powder for 30 min and then incubated with the specific antisera against PepMV or ToBRFV in the PBS-milk solution overnight at 4 °C. The samples were washed twice with PBS-T and the secondary antibody, goat anti-rabbit IgG [conjugated to Alexa Fluor 594 for PepMV, or to Alexa Fluor 488 for ToBRFV (Invitrogen, Carlsbad, CA, USA)], was added at a 1:1000 dilution in PBS and incubated for 1.5 h at 37 °C with agitation at 100 rpm. The samples were washed twice with PBS-T and then kept in PBS. Image acquisition was done using a Leica SP8 laser scanning microscope (Leica, Wetzlar, Germany), equipped with solid state lasers with 488 and 552 nm light, HC PL APO CS2 20x/0.75 objective (Leica, Wetzlar, Germany) and Leica Application Suite X software (LASX, Leica, Wetzlar, Germany). Green and red emission signals were detected with HyD (hybrid) detectors in the ranges of 500–540 and 560–660 nm, respectively.

### 4.7. Biological Assays for Fruit Infectivity Potential

Tomato plants *cv*. Ikram [a heterozygote, harboring a *Tm-2^2^* resistance allele], at the four-leaf stage, were subjected to biological assays using four inoculation modes: (A) Intact symptomatic fruits were rubbed onto leaves. (B) Rubbing leaves with intact symptomatic fruit contaminated hands, while injuring the inoculated leaf tissue. (C) Applying extracted fruit juice onto leaves. (D) Rubbing the extracted fruit juice onto leaves, while injuring leaf tissue. The four inoculation modes were carried out for each fruit, amounting to four plants per fruit. Three independent experiments with co-infected fruits were carried out. The three experiments were carried out at three different temperatures. **Exp. 1:** twenty-four fruits were used for the inoculation study carried out for twenty-three days, at 25–28 °C during the day and 16–19 °C at night. **Exp. 2:** fifteen fruits were used for the inoculation experiments carried out for forty-two days, at 16–20 °C during the day and 9–10 °C at night. **Exp. 3:** fifteen fruits were used for the inoculation experiments conducted for twenty-eight days at a controlled temperature of 24 ± 3 °C. An additional inoculation experiment, **Exp. 4,** with twenty-one fruits infected with ToBRFV only, was carried out for twenty-six days at 19–21 °C during the day and 11–13 °C at night. All fruits used for the four experiments were analyzed by ELISA. ELISA tests for fruits employed in Exp. 1 were conducted after the inoculation study, which was carried out with all of the symptomatic fruits. These ELISA results were confirmed by RT-PCR. The virus-inoculated plants were kept in a glasshouse and sprayed regularly with insecticides to prevent any infestation. All inoculated tomato plants were tested by ELISA and sampled plants were analyzed by western blot and RT-PCR tests.

## 5. Conclusions

ToBRFV and PepMV co-infected fruits were an effective inoculum source for viral disease spread in tomato plants. These results contributed to our understanding of ToBRFV and PepMV disease epidemiology and the importance of increased hygienic conduct awareness worldwide. Application of final fruit packaging procedures in proximity to the growing areas will prevent unnecessary fruit manipulations, thereby minimizing the risks of viral disease spread by consumers worldwide.

## 6. Patents

N/A.

## Figures and Tables

**Table 1:** Enzyme linked immunosorbent assay (ELISA) optical density (O.D.) results of pepino mosaic virus (PepMV) and/or tomato brown rugose fruit virus (ToBRFV) coat protein levels in symptomatic fruits subjected to inoculation experiments.

ELISA	ToBRFV				PepMV		
	Exp. 1 ^a,b^ (*n* = 24)	Exp. 2 (*n* = 15)	Exp. 3 (*n* = 15)	Exp. 4 (*n* = 21)	Exp. 1 ^a,b^ (*n* = 24)	Exp. 2 (*n* = 15)	Exp. 3 (*n* = 15)
**O.D. range**	0.067–2.776	0.265–0.936	0.246–0.929	0.121–1.214	0.874–3.44	0.844–1.856	0.607–1.801
**NR**	0.019	0.022–0.032	0.022–0.032	0.032–0.051	0.0314	0.006–0.021	0.006–0.021
**Infection ratio (%)**	100	100	100	100	79	100	100

^a^ Results were confirmed by RT-PCR; ^b^ ELISA test was performed after the inoculation assay; NR, negative reference; *n*, No. of fruits.

**Table 2:** Detection of pepino mosaic virus (PepMV) and tomato brown rugose fruit virus (ToBRFV) in leaf samples of tomato plants following four inoculation modes using enzyme linked immunosorbent assay (ELISA).

Treat.	ToBRFV ^a^								PepMV ^b^					
	Exp. 1 (*n* = 96)		Exp. 2 (*n* = 60)		Exp. 3 (*n* = 60)		Exp. 4 (*n* = 84)		Exp. 1 (*n* = 96)		Exp. 2 (*n* = 60)		Exp. 3 (*n* = 60)	
	Infect. Rate (%)	ELISA O.D. Range	Infect. Rate (%)	ELISA O.D. Range	Infect. Rate (%)	ELISA O.D. Range	Infect. Rate (%)	ELISA O.D. Range	Infect. Rate (%)	ELISA O.D. Range	Infect. Rate (%)	ELISA O.D. Range	Infect. Rate (%)	ELISA O.D. Range
**A**	0	0.01–0.03	0	0.01–0.02	0	0.02–0.07	0	0.01–0.03	0	0.01–0.03	0	0.01–0.02	0	0.04–0.08
**B**	0	0.01–0.03	0	0.01–0.02	0	0–0.07	0	0.01–0.03	0	0.01–0.03	0	0.01–0.03	0	0.05–0.06
**C**	29	0.04–0.95	13	0.07–1.67	20	0.32–1.4	5	0.31	5	0.08	20	0.08–0.44	7	0.52
**D**	88	0.1–1.09	47	0.25–1.17	93	0.11–0.83	67	0.09–0.66	67	0.1–2.26	47	0.32–0.84	80	0.13–1.65

^a^ ELISA optical density (O.D.) values of negative controls were 0.02–0.05; ^b^ ELISA O.D. values of negative controls were 0.01–0.03; *n*, total number of inoculated tomato plants; treat., treatment; infect., infection; **A**, rubbing leaves with intact symptomatic fruits; **B**, rubbing leaves with hands after touching intact symptomatic fruits; **C**, applying fruit juice from symptomatic fruits onto tomato leaves; **D**, rubbing leaves with fruit juice of symptomatic fruits.

## References

[1] Salem N., Mansour A., Ciuffo M., Falk B., Turina M. (2016). A new tobamovirus infecting tomato crops in Jordan. Arch. Virol..

[2] Luria N., Smith E., Reingold V., Bekelman I., Lapidot M., Levin I., Elad N., Tam Y., Sela N., Abu-Ras A. (2017). A New Israeli Tobamovirus Isolate Infects Tomato Plants Harboring Tm-22 Resistance Genes. PLoS ONE.

[3] Menzel W., Knierim D., Winter S., Hamacher J., Heupel M. (2019). First report of tomato brown rugose fruit virus infecting tomato in Germany. New Dis. Rep..

[4] Panno S., Caruso A., Davino S. (2019). First report of tomato brown rugose fruit virus on tomato crops in Italy. Plant Dis..

[5] Skelton A., Buxton-Kirk A., Ward R., Harju V., Frew L., Fowkes A. (2019). First report of Tomato brown rugose fruit virus in tomato in the United Kingdom. New Dis. Rep..

[6] Cambrón-Crisantos J.M., Rodríguez-Mendoza J., Valencia-Luna J.B., Rangel S.A., de Jesús García-Ávila C., López-Buenfil J.A. (2018). First report of Tomato brown rugose fruit virus (ToBRFV) in Michoacan, Mexico. Mex. J. Phytopathol..

[7] Ling K.-S., Tian T., Gurung S., Salati R., Gilliard A. (2019). First report of tomato brown rugose fruit virus infecting greenhouse tomato in the US. Plant Dis..

[8] Yan Z., Ma H., Han S., Geng C., Tian Y., Li X. (2019). First report of Tomato brown rugose fruit virus infecting tomato in China. Plant Dis..

[9] Smith E., Dombrovsky A. (2019). Aspects in Tobamovirus Management in Intensive Agriculture. Plant Pathol. Manag. Plant Dis. IntechOpen.

[10] Wright D., Mumford R. (1999). Pepino mosaic Potexvirus (PepMV): First records in tomato in the United Kingdom. Plant Disease Notice No. 89. Growth Horm. Igf Res..

[11] Van der Vlugt R.A.A., Stijger C.C.M.M., Verhoeven J.T.J., Lesemann D.E. (2000). First report of *Pepino mosaic virus* on tomato. Plant Dis..

[12] Lesemann D.E., Dalchow J., Winter S., Pfeilstetter E. (2000). Occurrence of Pepino mosaic virus in European tomato crops: Identification, etiology and epidemiology. Mitteilungen-Biologischen Bundesanstalt Fur Land Und Forstwirtschaft.

[13] French C., Bouthillier M., Bernardy M., Ferguson G., Sabourin M., Johnson R., Masters C., Godkin S., Mumford R. (2001). First report of Pepino mosaic virus in Canada and the United States. Plant Dis..

[14] Jordá C., Perez A.L., Martínez-Culebras P., Abad P., Lacasa A., Guerrero M. (2001). First report of Pepino mosaic virus on tomato in Spain. Plant Dis..

[15] Mumford R., Metcalfe E. (2001). The partial sequencing of the genomic RNA of a UK isolate of Pepino mosaic virus and the comparison of the coat protein sequence with other isolates from Europe and Peru. Arch. Virol..

[16] Roggero P., Masenga V., Lenzi R., Coghe F., Ena S., Winter S. (2001). First report of Pepino mosaic virus in tomato in Italy. Plant Pathol..

[17] Jones R.A.C., Koening R., Lesemann D.E. (1980). *Pepino mosaic virus*, a new potexvirus from pepino (*Solanum muricatum*). Ann. Appl. Biol..

[18] Mumford R.A., Jones R.A.C. (2005). *Pepino Mosaic Virus*; AAB Descriptions of Plant Viruses 411 No 11 Wellesbourne, UK: Association of Applied Biologists. http://www.dpvweb.net/dpv/showadpv.php?dpvno=411.

[19] Hanssen I., Paeleman A., Vandewoestijne E., Van Bergen L., Bragard C., Lievens B., Vanachter A.C.R.C., Thomma B.P.H.J. (2009). Pepino mosaic virus isolates and differential symptomatology in tomato. Plant Pathol..

[20] Sempere R.N., Gómez-Aix C., Ruíz-Ramón F., Gómez P., Hasiów-Jaroszewska B., Sánchez-Pina M.A. (2016). Pepino mosaic virus RNA-dependent RNA polymerase pol domain is a hypersensitive response-like elicitor shared by necrotic and mild isolates. Phytopathology.

[21] Ling K.S. (2007). Molecular characterization of two Pepino mosaic virus variants from imported tomato seed reveals high levels of sequence identity between Chilean and US isolates. Virus Genes.

[22] Maroon-Lango C., Guaragna M., Jordan R., Hammond J., Bandla M., Marquardt S. (2005). Two unique US isolates of Pepino mosaic virus from a limited source of pooled tomato tissue are distinct from a third (European-like) US isolate. Arch. Virol..

[23] Gómez P., Sempere R., Elena S.F., Aranda M.A. (2009). Mixed infections of Pepino mosaic virus strains modulate the evolutionary dynamics of this emergent virus. J. Virol..

[24] Hasiów-Jaroszewska B., Pospieszny H., Borodynko N. (2009). New necrotic isolates of Pepino mosaic virus representing the CH2 genotype. J. Phytopathol..

[25] Hanssen I.M., Thomma B.P. (2010). Pepino mosaic virus: A successful pathogen that rapidly evolved from emerging to endemic in tomato crops. Mol. Plant Pathol..

[26] Hasiów-Jaroszewska B., Borodynko N. (2012). Characterization of the necrosis determinant of the European genotype of pepino mosaic virus by site-specific mutagenesis of an infectious cDNA clone. Arch. Virol..

[27] Hasiów-Jaroszewska B., Borodynko N., Jackowiak P., Figlerowicz M., Pospieszny H. (2011). Single mutation converts mild pathotype of the Pepino mosaic virus into necrotic one. Virus Res..

[28] Hasiów-Jaroszewska B., Paeleman A., Ortega-Parra N., Borodynko N., Minicka J., Czerwoniec A., Thomma B.P., Hanssen I.M. (2013). Ratio of mutated versus wild-type coat protein sequences in P epino mosaic virus determines the nature and severity of yellowing symptoms on tomato plants. Mol. Plant Pathol..

[29] Duff-Farrier C.R., Bailey A.M., Boonham N., Foster G.D. (2015). A pathogenicity determinant maps to the N-terminal coat protein region of the Pepino mosaic virus genome. Mol. Plant Pathol..

[30] Chewachong G.M., Miller S.A., Blakeslee J.J., Francis D.M., Morris T.J., Qu F. (2015). Generation of an attenuated, cross-protective Pepino mosaic virus variant through alignment-guided mutagenesis of the viral capsid protein. Phytopathology.

[31] Lacasa A., Guerrero M., Hita I., Martinez M., ALcázar A., Cano A., Jordá C., Bielza P., Contreras J. (2003). Implication of bumble bees (Bombus spp.) on Pepino mosaic virus (PepMV) spread on tomato crops. Boletín de Sanidad Vegetal Plagas (España).

[32] Levitzky N., Smith E., Lachman O., Luria N., Mizrahi Y., Bakelman H., Sela N., Laskar O., Milrot E., Dombrovsky A. (2019). The bumblebee Bombus terrestris carries a primary inoculum of Tomato brown rugose fruit virus contributing to disease spread in tomatoes. PLoS ONE.

[33] Hanssen I.M., Mumford R., Blystad D.R., Cortez I., Hasiow-Jaroszewska B., Hristova D., Pagán I., Pereira A.M., Peters J., Pospieszny H. (2010). Seed transmission of Pepino mosaic virus in tomato. Eur. J. Plant Pathol..

[34] Cordoba-Selle M.D.C., Garcia-Randez A., Alfaro-Fernandez A., Jorda-Gutierrez C. (2007). Seed transmission of *Pepino mosaic virus* and efficacy of tomato seed disinfection treatments. Plant Dis..

[35] Dombrovsky A., Smith E. (2017). Seed Transmission of Tobamoviruses: Aspects of Global Disease Distribution. Adv. Seed Biol. Tech..

[36] EPPO (2019). European and Mediterranean Plant Protection Organization (EPPO) Global Database.

[37] Turco S., Golyaev V., Seguin J., Gilli C., Farinelli L., Boller T., Schumpp O., Pooggin M.M. (2018). Small RNA-omics for virome reconstruction and antiviral defense characterization in mixed infections of cultivated Solanum plants. Mol. Plant-Microbe Interact..

[38] Pagán I., del Carmen Córdoba-Sellés M., Martínez-Priego L., Fraile A., Malpica J.M., Jordá C., García-Arena F. (2006). Genetic structure of the population of Pepino mosaic virus infecting tomato crops in Spain. Phytopathology.

[39] Davino S., Davino M., Bellardi M.G., Agosteo G.E. (2008). Pepino mosaic virus and Tomato chlorosis virus causing mixed infection in protected tomato crops in Sicily. Phytopathol. Mediterr..

[40] Gómez P., Sempere R., Amari K., Gómez-Aix C., Aranda M. (2010). Epidemics of Tomato torrado virus, Pepino mosaic virus and Tomato chlorosis virus in tomato crops: Do mixed infections contribute to torrado disease epidemiology?. Ann. Appl. Biol..

[41] Iacono G., Hernandez-Llopis D., Alfaro-Fernandez A., Davino M., Font M., Panno S., Galipenso L., Rubio L., Davino S. (2015). First report of Southern tomato virus in tomato crops in Italy. New Dis Rep..

[42] Koenig R. (1981). Indirect ELISA Methods for the Broad Specificity Detect. Plant Viruses. J. Gen Virol..

[43] Reingold V., Lachman O., Belausov E., Koren A., Mor N., Dombrovsky A. (2016). Epidemiological study of *Cucumber green mottle mosaic virus* in greenhouses enables reduction of disease damage in cucurbit production. Ann. Appl. Biol..

